# N-Acetyl-L-Cysteine Protects Organ Function After Hemorrhagic Shock Combined With Seawater Immersion in Rats by Correcting Coagulopathy and Acidosis

**DOI:** 10.3389/fphys.2022.831514

**Published:** 2022-03-22

**Authors:** Yiyan Liu, Yu Zhu, Zisen Zhang, Daiqin Bao, Haoyue Deng, Liangming Liu, Tao Li

**Affiliations:** State Key Laboratory of Trauma, Burns and Combined Injury, Department of Shock and Transfusion, Research Institute of Surgery, Army Medical Center, Army Medical University, Chongqing, China

**Keywords:** seawater immersion, hemorrhagic shock, oxidative stress seawater, immersion, organ function, acidosis, coagulopathy

## Abstract

**Background:**

The mortality of trauma combined with seawater immersion is higher than that of land injury, however, research on how to treat this critical case and which treatments to adopt is lacking.

**Methods:**

The effect of the thiol compound, N-acetyl-L-Cysteine (NAC), on survival, acidosis, coagulopathy, vital signs, oxidative stress, and mitochondrial and multi-organ function was assessed in a rat model of hemorrhagic shock combined with seawater immersion (Sea-Shock).

**Results:**

Hemorrhagic shock combined with seawater immersion caused a severe lethal triad: multi-organ impairment, oxidative stress, and mitochondrial dysfunction. NAC (30 mg/kg) with lactated Ringer’s (LR) solution (2 × blood volume lost) significantly improved outcomes compared to LR or hetastarch (HES 130/0.4) alone. NAC significantly prolonged survival time to 52.48 ± 30.09 h and increased 72 h survival rate to 11/16 (68%). NAC relieved metabolic acidosis and recovered the pH back to 7.33. NAC also restored coagulation, with APTT, PT, and PT-INR decreased by 109.31, 78.09, and 73.74%, respectively, while fibrinogen level increased 246.23% compared with untreated Sea-Shock. Administration of NAC markedly improved cardiac and liver function, with some improvement of kidney function.

**Conclusion:**

The addition of NAC to crystalloid resuscitation fluid alleviated oxidative stress, restored redox homeostasis, and provided multi-organ protection in the rats after Sea-Shock. NAC may be an effective therapeutic measure for hemorrhagic shock combined with seawater immersion.

## Introduction

With the rapid development of the economy and globalization, marine operation and local wars at sea are escalating, unfortunately accompanied by increasing occurrence of marine accidents, which cause huge loss of life and property. Seawater has several characteristics such as low temperature, alkalinity, and high concentrations of sodium, potassium, calcium, magnesium, and other electrolytes (Wei et al., 2016). Our previous studies found that hemorrhagic shock combined with seawater immersion induced several complicated and fatal pathological features including high incidence of hypothermia, coagulation disorder, and acidosis, as well as electrolyte disorder and high incidence of multiple organ dysfunction, which resulted in high mortality (Liu Y. et al., 2020; Zhu et al., 2020; Lin et al., 2021). However, research on how to treat hemorrhagic shock combined with seawater immersion and what drugs to adopt are insufficient at present.

Numerous studies demonstrated that oxidative stress was one of the major pathophysiological characteristics following hemorrhagic shock (Lei et al., 2020). Overloaded reactive oxygen species (ROS) will cause nucleus, cell membrane, and organelle damage, and even induce cell death. Ma et al. (2017) highlighted that seawater due to its characteristics could aggravate oxidative stress injury in hemorrhagic shock, and a similar conclusion that the mortality of trauma combined with seawater immersion is several times higher than that of land injuries was drawn by Liu K. L. et al. (2020). NAC, namely N-acetyl-L-Cysteine, a thiol compound with the relative molecular mass of 163.2, is the precursor of L-cysteine and glutathione, a strong antioxidant (Aldini et al., 2018). There are some reports that NAC plays a therapeutic role in hemorrhagic shock, ulcerative colitis, and cardiac ischemia reperfusion injury due to its properties (Lee et al., 2013; Moreira et al., 2016; Pei et al., 2018; Sivasinprasasn et al., 2019; Masnadi Shirazi et al., 2021). Lee et al. (2013) showed that NAC could attenuate acute lung and kidney injury after hemorrhagic shock in rat. Similar conclusions were drawn in the studies of Lee et al. (2013) and Moreira et al. (2016). Current therapeutic measures recommended for hemorrhagic shock combined with seawater immersion have not referred to anti-oxidation and it is unclear whether NAC can protect multi-organ function in hemorrhagic shock combined with seawater immersion via anti-oxidation.

Therefore, our study constructed models of hemorrhagic shock rats combined with 15°C seawater immersion to investigate the effects of NAC on animal survival, acidosis, coagulation disorders, vital signs, multi-organ function, oxidative stress, and mitochondrial function, so as to provide an experimental basis for applicable therapeutic measures for those who accidentally fall overboard or are wounded in marine accidents.

## Materials and Methods

### Ethical Approval of the Study Protocol

All animal experiments were carried out in strict accordance with the principles of the “Guide for the Care and Use of Laboratory Animals” (eighth edition, 2011, National Academies Press, Washington, DC, United States) and approved by the Laboratory Animal Welfare and Ethics Committee of Third Military Medical University (Chongqing, China). None of the authors are members of this committee.

### Animal Model and Experimental Protocol

The animals were randomly distributed into the following groups:

- LR group: This group of animals was submitted to hemorrhagic shock combined with seawater immersion and treated with lactated Ringer’s (LR) solution (2 × volume of blood loss).

- HES (130/0.4) group: This group of animals was submitted to hemorrhagic shock combined with seawater immersion and treated with hetastarch (HES) solution (2 × volume of blood loss).

- LR + N-acetyl-L-Cysteine group: This group of animals was submitted to hemorrhagic shock combined with seawater immersion and treated with LR solution (2 × volume of blood loss) associated with NAC (30 mg/kg).

- Sham group: This group of animals was anesthetized and underwent all surgical procedures except blood drawing and seawater immersion.

**FIGURE 1:**
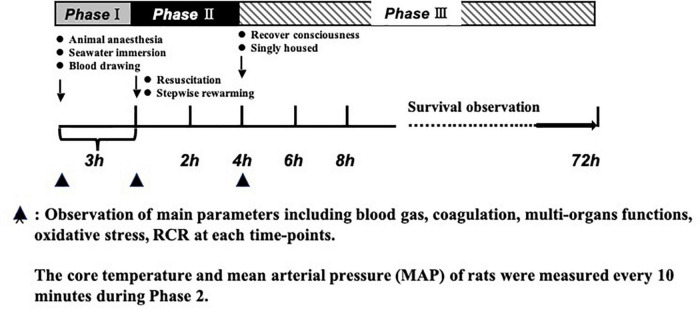
Timeline of the experimental protocol. Phase 1: establishment of model of hemorrhagic shock combined with seawater immersion. Phase II: administration of fluid (2 × volume of blood loss) and stepwise rewarming. Phase III: observation period including animal survival, coagulation parameters, multi-organ function, MDA, ROS, and RCR. ▲ Observation of main parameters including blood gas, coagulation, multi-organ functions, oxidative stress, and RCR at each time point.

A total of 184, half male and half female, Sprague-Dawley rats (SD) (200 ± 20 g) aged 12–14 weeks were used in this study. Experiment animals were raised in a controlled temperature (22 ± 2°C) and a 12 h light/dark period. To facilitate total blood loss measurements from rat weight, animals were fasted 12 h before the experiment, but with *ad libitum* access to water containing glucose for energy. The rats were anesthetized with sodium pentobarbital (30 mg/kg, intraperitoneal), which generally maintained anesthesia for 4 h. Then, we added 1.5–3 mg of sodium pentobarbital depending on the state of the rats, to guarantee the rats had no response to a needle stimulus. Due to the sedative and analgesic effects of sodium pentobarbital, no additional analgesia (topical or systemic) was administered. We used aseptic techniques for surgical procedures, including skin incision, catheterization, and suture (with 2-0 stitches to close the muscle and then the skin). After suturing, the rats were given intramuscular injections of penicillin and streptomycin (100,000 units of streptomycin and penicillin per kilogram). Rats were able to breathe spontaneously without mechanical ventilation. The carotid artery and jugular vein were catheterized with a polyethylene catheter (outer diameter, 0.965 mm; inner diameter, 0.58 mm) and the temperature probe was placed concomitant with the carotid artery for core body temperature monitoring. Carotid artery intubation was used for monitoring blood pressure with an electronic sphygmomanometer (-500-500 KPA/0.4, HaoGan, China), and blood extraction and jugular vein intubation was used for drug administration. To prevent clot formation, the carotid artery catheter was filled with normal (physiological) saline (0.9%) containing 30 U/mL of heparin. Then the rats were vertically immersed in seawater at 15°C, with armpits above the surface to maintain normal breathing. Next, we began to draw blood. The carotid blood pressure was reduced to 40 mmHg within 20 min and remained stable at 40 mmHg for 2 h. For the whole process, rats were immersed in seawater. The experiments were conducted in three parts. Phase I was the model stage, which has been described above. Phase II was the resuscitation stage. Based on previous pilot results from our lab, we adopted a stepwise rewarming protocol. We pulled rats out of seawater, placed them in 34°C incubators for 2 h immediately and administrated fluid (2 × volume of blood loss) via the jugular vein catheter (at a rate of 25 mL/h). Then, we replaced rats in 37°C incubators for 2 h. Phase III was the observation phase. Parameters including coagulation, vital organs function, ROS, MDA, RCR, and survival time were measured. During the period of survival observation, all catheters were removed, and incisions were closed. The rats were singly housed during the 72-h monitoring. If the rats survived over 72 h, they were euthanized by overdose of sodium pentobarbital (Figure 1).

### Malondialdehyde Assay

Malondialdehyde was measured using a malondialdehyde (MDA) assay kit (catalog no. A003-1; Nanjing Jiancheng Technology Company, China). The rats were killed and the liver, kidney, heart, superior mesenteric artery, lung, and blood serum were collected. Tissues were cut into small pieces, washed with PBS to remove blood, then dried with filter paper, and transferred to an EP tube. Lysis solution was added to the tube and the volume of the lysate solution was 10-fold the weight of the tissues. EP tubes were placed in ice for 50 min and then were centrifugated at 12,000 *g* at 4°C for 15 min. The supernatant was taken to be tested. A 0.1 mL sample and 0.2 mL of MDA working solution were mixed and then heated at 100°C for 15 min. After cooling to room temperature, the mixture was centrifuged at 1,000 *g* for 10 min. The 200 μl supernatant was used for measurement at 532 nm with a microplate analyzer. MDA concentration was then obtained according to the standard curve. A BCA Protein Assay Kit (Sigma) was used for measuring the total protein of tissues. Results were normalized with the protein level (*n* = 8/group).

### Reactive Oxygen Species Assay

Reactive oxygen species was measured using a reactive oxygen species (ROS) assay kit (catalog no. E004; Nanjing Jiancheng Technology Company, China). The tissue collection and processing were identical to the MDA assay. The precipitation was suspended by diluted DCFH-DA (100 μM) and incubated at 37°C for 1 h. Then the sample was centrifuged for 10 min at 1,000 *g*, and the supernatant was removed to collect the precipitation, which was then washed twice with PBS. The precipitation was centrifuged for 10 min at 1,000 *g* and collected for fluorescence detection. According to the FITC fluorescence detection conditions, the results were expressed as the value of fluorescence intensity (*n* = 8/group).

### Respiratory Control Ratio Assay

The Respiratory Control Ratio (RCR) is an indicator of the state of mitochondrial coupling, which is related to mitochondrial functionality. Decreasing RCR reflects compromised mitochondrial efficiency in adenosine triphosphate production (Gupte et al., 2016; Fadel et al., 2018; Courtes et al., 2020). Mitochondrial function was measured as described in our previous study. Briefly, tissues were placed in pre-cooled isolation buffer (sucrose 0.25 M, Na_2_EDTA 0.1 mM, Tris 0.01 M, pH 7.6), and blood was removed as much as possible. After homogenizing and centrifuging the tissues at 1,600 *g* for 12 min at 4°C, the supernatant was further centrifuged twice at 25,000 *g* for 15 min at 4°C. The precipitation was resuspended with an isolation buffer. The concentration of mitochondrial protein was measured by the Lowry method: 30°C reaction buffer (Tris 0.2 M, pH 7.6, KCl 15 mM, KH_2_PO_4_ 15 mM, Na_2_EDTA 1 mM, MgCl_2_ 5 mM, and sucrose 0.25 M) was added into the chamber until equilibrium. Then, mitochondrial mixture, sodium malate, sodium glutamate, and adenosine diphosphate were added in turn. The rate of oxygen consumption was determined by a mitochondrial function analyzer (MT200; Strathkelvin) (*n* = 8/group).

### Coagulation Parameters and Cardiac, Liver, and Kidney Function Measurement

A total of 2 ml of blood (sodium citrate: blood = 1:9) was collected and centrifuged at 1,500 *g* for 15 min to measure the coagulation function parameters using ACL TOP 700 before and after Sea-Shock and at the end of Phase II. Thrombin time (TT), prothrombin time (PT), international normalized ratio of prothrombin time (PT-INR), activated partial prothrombin time (APTT), cardiac function: cardiac troponin (TnT), liver function: aspartate aminotransferase (AST) and alanine aminotransferase (ALT), kidney function: blood urea nitrogen (BUN), and serum creatinine (Crea) were measured by an automatic biochemistry analyzer in the clinical laboratory of Daping hospital before and after Sea-Shock and at the end of Phase II (*n* = 8/group).

### Blood Gas and Core Temperature

Blood gas was measured by a blood gas analyzer (ABL800). The core temperature and mean arterial pressure (MAP) of rats were measured every 10 min during Phase II (*n* = 8/group).

### Survival Parameters

Sixty rats received carotid artery and vein catheterization, bleeding, and immersing procedures as previously detailed. At the end of Phase II, catheters were removed, incisions closed, and rats were further observed for 72 h, the survival time and 72 h survival rate were finally calculated. The rats were put back into their cages, singly housed for 72-h monitoring. Mortality was defined by no heartbeat or absence of respiration, and rats that survived over 72 h were euthanized by overdose of sodium pentobarbital (*n* = 16/group).

### Statistical Analyses

Data were presented as the mean ± SD of n observations. Statistical differences were analyzed by repeated measure one-way or two-way ANOVA analyses, followed by the *post hoc* Tukey test (SPSS 17.0, SPSS Incorporated, Chicago, IL, United States). Before the application of ANOVA, the distribution of data was assessed using the Kolmogorov–Smirnov test. The results showed that all parametric data were normally distributed. Survival time and survival prevalence were individually analyzed by mean ± SD, Kaplan–Meier survival analyses, and the log rank test. *p* < 0.05 (two-tailed) was considered significant.

## Results

### Effects of N-Acetyl-L-Cysteine on Coagulation Disorders and Acidosis Induced by Hemorrhagic Shock Combined With Seawater Immersion in Rats

#### Acidosis

Rats presented severe acidosis following hemorrhagic shock combined with seawater immersion, which was embodied in a PH decrease to 7.04 (Table 1). LR solution or HES solution used alone just corrected the acidosis slightly and pH was increased to 7.24 and 7.23, respectively, at the end of Phase II (Table 1). Administration of LR solution with addition of NAC (30 mg/kg) significantly antagonized acidosis, which was reflected in the raised value of pH to 7.33 at the end of Phase II (Table 1), almost recovering to the normal level. The lactic acid level of arterial blood was further observed on account of its great significance in predicting the risk of death in critical patients. The results showed that the arterial lactate level was significantly increased after hemorrhagic shock combined with seawater immersion and simple crystalloid liquid LR or colloidal liquid used alone failed to restore the arterial lactate to a normal level, while the level of arterial lactate after administration of LR solution with addition of NAC was significantly decreased to a normal level (Table 1). Similarly, the values of pCO_2_ and pO_2_ in the NAC group were significantly improved (Table 1).

**TABLE 1:** Changes of blood gas.

Group	Baseline	End of phase 1	End of fluid infusion	End of 34°C recovery	End of Phase II
pH					
LR	7.37 ± 0.22	7.04 ± 0.82^∧∧∧^	7.14 ± 0.31	7.21 ± 0.44	7.24 ± 0.63
HES	7.37 ± 0.22	7.04 ± 0.82^∧∧∧^	7.15 ± 0.60	7.20 ± 0.17	7.23 ± 0.53
NAC	7.37 ± 0.22	7.04 ± 0.82^∧∧∧^	7.19 ± 0.40^#^	7.25 ± 0.28*	7.33 ± 0.40^##^ ***
Lac					
LR	2.80 ± 0.68	10.10 ± 1.40^∧∧∧^	8.26 ± 2.32	6.70 ± 1.79	6.40 ± 0.8
HES	3.06 ± 0.77	10.3 ± 1.99^∧∧∧^	8.33 ± 2.11	6.16 ± 2.93	6.33 ± 2.63
NAC	3.20 ± 0.39	10.16 ± 1.89^∧∧∧^	6.09 ± 1.57^#^*	4.24 ± 1.75*	5.29 ± 2.53^##^ **
pO_2_, mmHg					
LR	143.63 ± 19.70	81.13 ± 7.75^∧∧∧^	91.13 ± 6.22	99.25 ± 6.54	115.38 ± 12.34
HES	145.25 ± 14.34	76.5 ± 10.42^∧∧∧^	84.38 ± 10.57	92.38 ± 9.21	101.88 ± 6.81
NAC	156.88 ± 18.28	80.25 ± 9.36^∧∧∧^	90.13 ± 6.13	121.0 ± 18.35^##^ ***	130.13 ± 13.11^#^ ***
pCO_2_, mmHg					
LR	49.48 ± 3.53	33.44 ± 2.55^∧∧∧^	34.88 ± 4.81	36.80 ± 4.89	40.25 ± 4.87
HES	49.74 ± 4.55	32.21 ± 2.82^∧∧∧^	35.23 ± 3.94	38.04 ± 5.02	34.91 ± 5.23
NAC	49.55 ± 4.01	32.33 ± 4.19^∧∧∧^	37.46 ± 4.15	40.93 ± 3.24	43.60 ± 2.87***

Data presented as mean ± standard deviation (n = 8/group). LR, Lactated Ringer’s; HES, Hetastarch; NAC, LR solution with the addition of NAC (30 mg/kg). ^∧∧∧^P < 0.001, Sea-Shock group versus baseline; ^#^P < 0.05, ^##^P < 0.01, LR group versus NAC group; *P < 0.05, **P < 0.01, ***P < 0.001, HES group versus NAC group.

#### Coagulation Function

**FIGURE 2:**
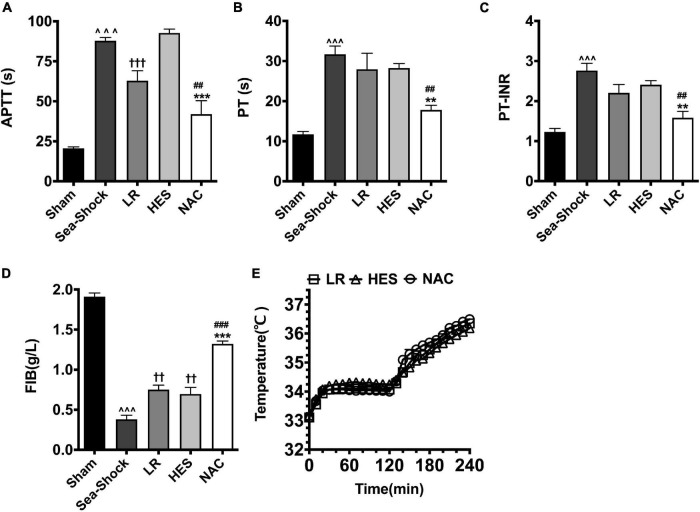
Effects of LR solution, HES solution, and LR solution with the addition of NAC (30 mg/kg) on coagulation function and core temperature. **(A)** APTT; **(D)** FIB; **(B)** PT; **(C)** PT-INR; **(E)** temperature. LR, Lactated Ringer’s; HES, Hetastarch; NAC, LR solution with the addition of NAC (30 mg/kg). Data presented as mean ± standard deviation (*n* = 8/group). ^∧∧∧^*P* < 0.001, Sea-Shock group versus baseline; ^††^*P* < 0.01, ^†††^*P* < 0.001, LR group or HES group versus Sea-Shock group; ^##^*P* < 0.01, ^###^*P* < 0.001, LR group versus NAC group; **P < 0.01, ***P < 0.001, HES group versus NAC group.

Coagulation function was disturbed significantly following hemorrhagic shock combined with seawater immersion, which indicated that APTT, PT, and PT-INR (Figures 2A–C) were significantly increased and FIB (Figure 2D) was significantly decreased. Following LR solution or HES solution used alone, APTT, PT, and PT-INR were slightly decreased, and FIB was also just slightly increased at the end of Phase II. APTT, PT, and PT-INR (Figures 2A–C) were decreased by 109.31, 78.09, and 73.74%, respectively, and FIB (Figure 2D) was increased by 246.23% at the end of Phase II, as compared with the Sea-Shock group, which demonstrated that NAC could restore coagulation function significantly. The core temperature of rats in each group was able to increase (Figure 2E).

### Effects of N-Acetyl-L-Cysteine on Vital Organ Dysfunction Induced by Hemorrhagic Shock Combined With Seawater Immersion in Rats

**FIGURE 3:**
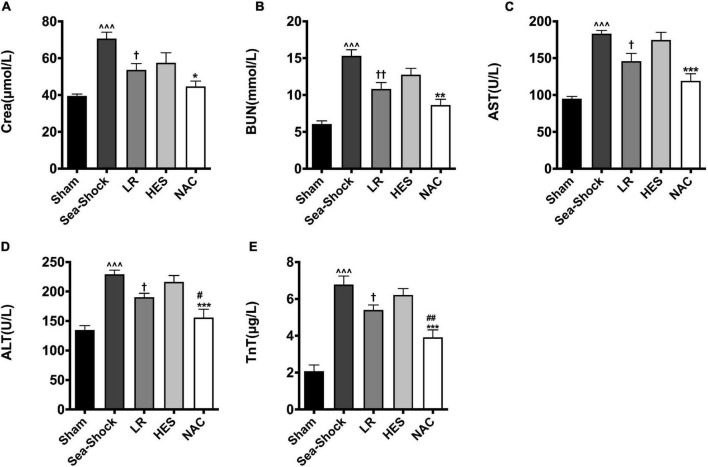
Effects of LR solution, HES solution, and LR solution with the addition of NAC (30 mg/kg) on cardiac, liver, and kidney function. **(A)** Crea; **(B)** BUN; **(C)** AST; **(D)** ALT; **(E)** TnT. LR, Lactated Ringer’s; HES, Hetastarch; NAC, LR solution with the addition of NAC (30 mg/kg). Data presented as mean ± standard deviation (*n* = 8/group). ^^^*P* < 0.001, Sea-Shock group versus baseline; ^†^*P* < 0.05, ^††^*P* < 0.01, LR group or HES group versus Sea-Shock group; ^#^*P* < 0.05, ^##^*P* < 0.01, LR group versus NAC group; *P < 0.05, **P < 0.01, ***P < 0.001, HES group versus NAC group.

The present results showed that hemorrhagic shock combined with seawater immersion led to severe organ impairment, embodied in the significantly elevated cardiac damage parameter (TnT), liver damage parameters (AST and ALT), and kidney damage parameters (Crea and BUN) (Figures 3A–E). LR solution or HES solution used alone could not largely alleviate the damage of the heart, liver, and kidney, while multiple organ function was largely recovered following NAC administration, especially liver and heart function.

### Effects of N-Acetyl-L-Cysteine on Mean Arterial Pressure and Animal Survival Following Hemorrhagic Shock Combined With Seawater in Rats

**FIGURE 4:**
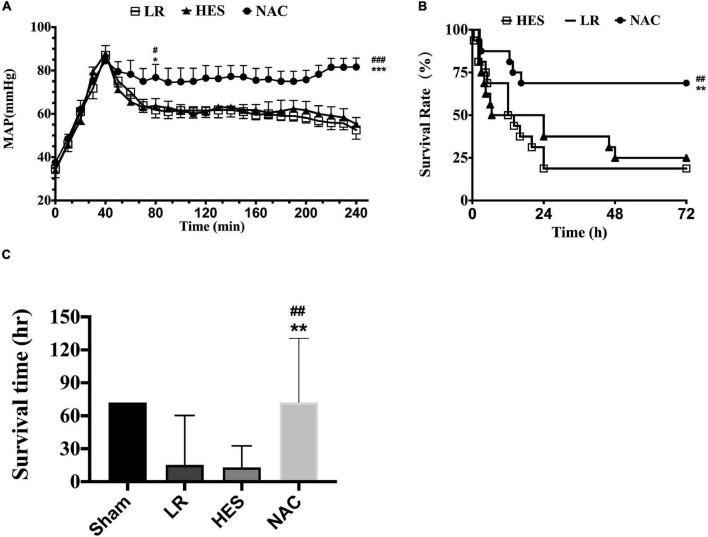
Effects of LR solution, HES solution, and LR solution with the addition of NAC (30 mg/kg) on MAP, survival time, and 72-h survival rate. **(A)** MAP (*n* = 8/group); **(C)** survival time (*n* = 16/group); **(B)** 72-h survival rate (*n* = 16/group). LR, Lactated Ringer’s; HES, Hetastarch; NAC, LR solution with the addition of NAC (30 mg/kg). Data presented as mean ± standard deviation (*n* = 16/group). ^#^*P* < 0.05, ^##^*P* < 0.01, ^###^*P* < 0.001, LR group versus NAC group; *P < 0.05, **P < 0.01, ***P < 0.001, HES group versus NAC group.

The mean arterial pressure (MAP) fell to 40 mmHg after hemorrhagic shock combined with seawater immersion and could be raised to 80 mmHg approximately in all groups at the end of infusion. However, the MAP failed to maintain at this level with LR solution or HES solution used alone and decreased to 50 mmHg rapidly. The MAP was maintained at 80 mmHg in the NAC group (Figure 4A). As for animal survival, the 24-h death rate with LR solution or HES solution used alone was 11 and 10, respectively, while the 24-h death rate was just 5 in the NAC group (Figure 4B), which was significantly lower. LR solution with the addition of NAC (30 mg/kg) significantly improved the survival time and 72-h survival rate to 52.48 ± 7.283 h and 11/16 (68%) and the 95% confidence interval ranged from 38.207 to 66.757 h (Figure 4B). The 72-h survival rate was 4/16 (25%), the 72-h survival time was 28.844 ± 7.164 h, and the 95% confidence interval ranged from 14.802 to 42.885 h in the LR group. The 72-h survival rate was 3/16 (18.75%), the 72-h survival time was 22.75 ± 6.175 h, and the 95% confidence interval ranged from 10.648 to 34.852 h in the HES group (Figure 4C).

### The Mechanism of N-Acetyl-L-Cysteine Protecting the Lethal Triad Following Hemorrhagic Shock Combined With Seawater Immersion

#### Oxidative Stress

**FIGURE 5:**
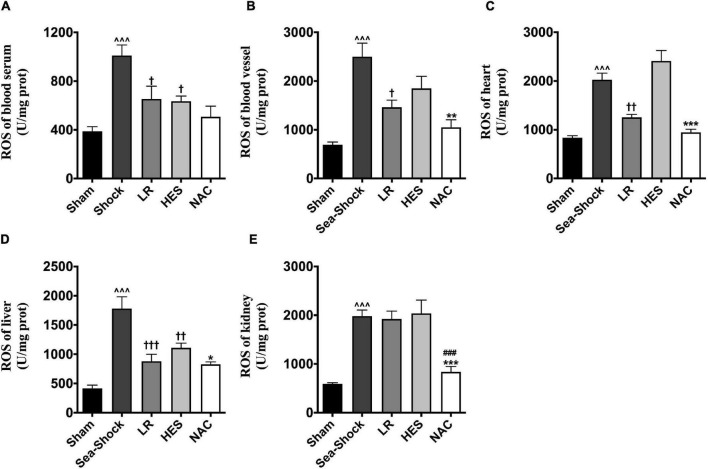
Effects of LR solution, HES solution, and LR solution with the addition of NAC (30 mg/kg) on ROS. **(A)** ROS of blood vessel; **(B)** ROS of heart; **(C)** ROS of kidney; **(D)** ROS of liver; **(E)** ROS of lung. LR, Lactated Ringer’s; HES, Hetastarch; NAC, LR solution with the addition of NAC (30 mg/kg). Data presented as mean ± standard deviation (*n* = 8/group). ^∧∧∧^*P* < 0.001, Sea-Shock group versus baseline; ^†^*P* < 0.05, ^††^*P* < 0.01, ^†††^*P* < 0.001, LR group or HES group versus Sea-Shock group; ^###^*P* < 0.001, LR group versus NAC group; *P < 0.05, **P < 0.01, ***P < 0.001, HES group versus NAC group.

**FIGURE 6:**
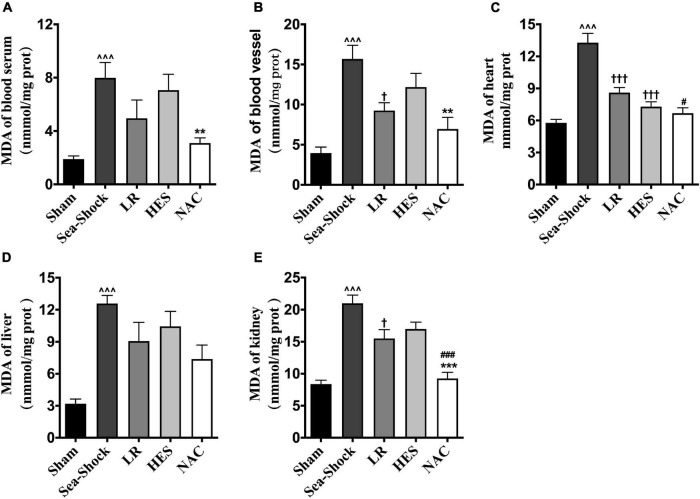
Effects of LR solution, HES solution, and LR solution with the addition of NAC (30 mg/kg) on MDA. **(C)** MDA of heart; **(A)** MDA of blood serum; **(B)** MDA of blood vessel; **(E)** MDA of kidney; **(D)** MDA of liver. LR, Lactated Ringer’s; HES, Hetastarch; NAC, LR solution with the addition of NAC (30 mg/kg). Data presented as mean ± standard deviation (*n* = 8/group). ^∧∧∧^*P* < 0.001, Sea-Shock group versus baseline; ^†^*P* < 0.05, ^†††^*P* < 0.001, LR group or HES group versus Sea-Shock group; ^#^*P* < 0.05, ^###^*P* < 0.001, LR group versus NAC group; **P < 0.01, ***P < 0.001, HES group versus NAC group.

Reactive oxygen species (ROS) and malondialdehyde (MDA) in plasma and heart, kidney, and liver were significantly increased following hemorrhagic shock combined with seawater immersion (Figures 5A–E, 6A–E). Simple LR solution or HES solution could decrease the level of ROS and MDA in plasma and multiple organs, and LR solution worked better than HES solution. LR solution with the addition of NAC (30 mg/kg) could significantly attenuate the ascending tendency of ROS and MDA. The level of ROS in the heart and kidney was decreased by 53.34 and 57.78%, respectively, and the level of MDA in the heart and kidney was decreased by 49.75 and 55.89%, respectively, (Figures 5C,E, 6C,E).

#### Mitochondrial Function

**FIGURE 7:**
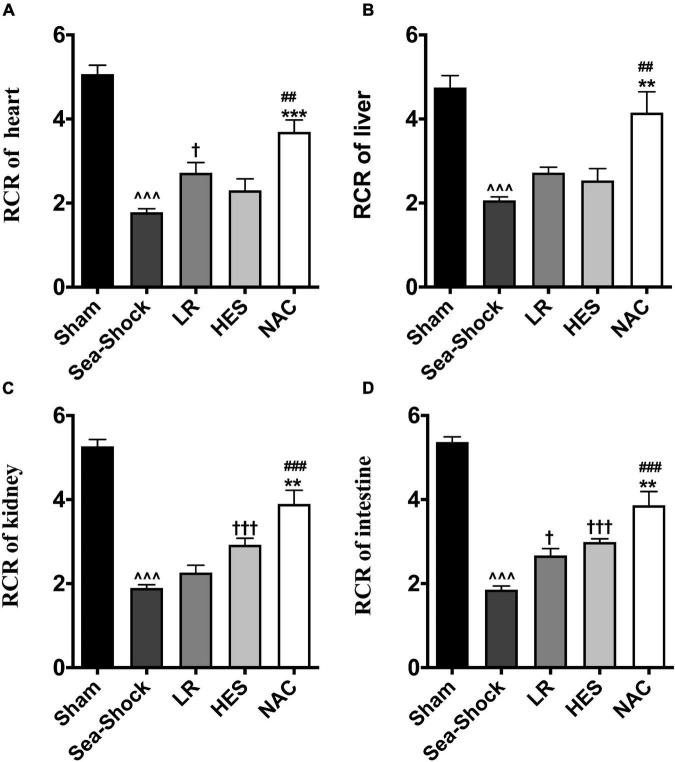
Effects of LR solution, HES solution, and LR solution with the addition of NAC (30 mg/kg) on mitochondrial function. **(A)** RCR of heart; **(D)** RCR of intestine; **(C)** RCR of kidney; **(B)** RCR of liver. RCR, respiratory control rate; LR, Lactated Ringer’s; HES, Hetastarch; NAC, LR solution with the addition of NAC (30 mg/kg). Data presented as mean ± standard deviation (*n* = 8/group). ^∧∧∧^*P* < 0.001, Sea-Shock group versus baseline; ^†^*P* < 0.05, ^†††^*P* < 0.001, LR group or HES group versus Sea-Shock group; ^##^*P* < 0.01, ^###^*P* < 0.001, LR group versus NAC group; **P < 0.01, ***P < 0.001, HES group versus NAC group.

The respiratory control rate of the heart, intestine, kidney, and liver was significantly decreased following hemorrhagic shock combined with seawater immersion (Figures 7A–D). LR solution or HES solution used alone increased the respiratory control rate slightly while the respiratory control rate of the heart, intestine, kidney, and liver was significantly increased following administration of LR solution with the addition of NAC (30 mg/kg) (Figures 7A–D), which indicated that NAC could preserve mitochondrial function to some degree.

## Discussion

With the increasing incidence of marine accidents, growing attention has been given to the effective measure for emergency rescue, especially those accompanied with hemorrhagic shock. Our previous studies found that hemorrhagic shock could generate multi-organ dysfunction (MODS) in rats, which further deteriorated in rats following hemorrhagic shock combined with seawater immersion (Liu Y. et al., 2020; Zhu et al., 2020; Lin et al., 2021). We speculated that low temperature seawater immersion aggravated the lethal triad, which might play a crucial role in the development of MODS. First of all, seawater is of high thermal conductivity. Therefore, cold seawater decreases core temperature rapidly. In addition, hypothermia exerts a significant influence on coagulation, embodied in the fact that thrombin activity was decreased, the internal and external coagulation pathways were impaired, platelets in liver and spleen were increased, and platelets in blood were decreased. Hypothermia also induces platelets to release heparin cofactor, resulting in excessive anticoagulation. Cold seawater immersion can also cause metabolic acidosis (Buse et al., 2017; Kander and Schött, 2019). Therefore, how to protect organs and recover their function effectively following hemorrhagic shock and low temperature seawater immersion need be studied further.

N-acetyl-L-Cysteine (NAC) is a thiol-containing compound with antioxidant and anti-inflammatory properties, exhibiting effects on microcirculation (Saad et al., 2012). NAC acts directly as an antioxidant, by deactivating reactive oxygen species, or indirectly, by replacing intracellular glutathione stores. Its oral dosage has been used clinically as a mucolytic agent to reduce sputum viscosity and allow sputum to be easily dissolved and discharged. In addition, it is also used clinically in drug poisoning, such as acetaminophen and heavy metals, radiation injury, AIDS, and cancer because of its powerful antioxidant action via increasing the level of glutathione (Roederer et al., 1993; Gillissen et al., 1997; Hao et al., 2008; Aggarwal et al., 2020; Kim et al., 2020). NAC has also been studied to treat COVID-19 recently (Ibrahim et al., 2020). Some studies reported that NAC could attenuate lung injury via decreasing cell death (Saad et al., 2012) and attenuate inflammatory response and acute lung injury (Lee et al., 2013). Moreira et al. (2016) reported that NAC is a promising drug for combining with fluid resuscitation to attenuate the kidney injury associated with hemorrhagic shock via reducing oxidative stress and apoptosis. However, there have been few reports regarding the role of NAC in protection of hemorrhagic shock combined with seawater immersion, which has higher mortality than hemorrhagic shock on land.

Lactated Ringer’s solution combined with corticotropin, amphotericin B, norepinephrine, tetracycline, etc., will precipitate effects. In this study, after adding NAC into lactated Ringer’s solution, the solution was still clear and had no precipitation. Besides, Lee et al. (2013) and Moreira et al. (2016) have pointed out that NAC higher than 100 mg/kg has a therapeutic effect on hemorrhagic shock. A study found that 30 mg/kg of NAC has a therapeutic effect on hemorrhagic shock combined with seawater immersion. Therefore, NAC has the potential to be adopted as a safe and effective drug in Sea-Shock. According to our experiment results, we propose the mechanism of how NAC corrected coagulopathy and acidosis as follows. NAC might recover coagulation via its properties. At present, oxidative stress has been highlighted in the initiation and progression of coagulation. Studies from Schafer et al. (2003) pointed that oxidative stress was important in the process of thrombosis by observation that ROS upregulated the expression level of PAI-I in endothelial cells, and enhanced PAI-1 expression in vasculature promotes prothrombotic phenotype and atherosclerosis in ApoE^–/–^ mice, which is consistent with the improved coagulation parameters following NAC administration in this study. NAC might also alleviate acidosis via its antioxidant capability. Low temperature seawater immersion combined with hemorrhagic shock led to a sharp decrease in circulating blood volume, and thereby perfusion of tissues and organs became seriously insufficient. In the meanwhile, the disturbance of microcirculation further aggravated the ischemia and hypoxia of tissues following Sea-Shock. When the rats were in an anoxic state, anaerobic fermentation in tissue cells were thus enhanced, causing the accumulation of acid products (Gupte et al., 2016). Some studies demonstrated that oxidative stress was a result of hypoxia and acidosis but whether improved oxidative stress could correct acidosis was unclear (Aryal et al., 2020). Our results showed that LR solution with addition of NAC (30 mg/kg) almost recovered the PH back to the normal level, which was better than LR solution or HES solution used alone. We analyzed the above changes and speculated that improved mitochondrial function might play an important role in this process, in which aerobic respiration was increased, lactic acid production was decreased, and eventually metabolic acidosis was alleviated. Mitochondria are the main source of oxygen radicals. In normal conditions, endogenous scavengers (vitamin C, vitamin E, glutathione, etc.) keep the redox balance. In the situation of hemorrhagic shock combined with seawater immersion, the redox balance is disrupted and a large number of radicals are synthesized, attacking mitochondria (and membrane, nucleic acid, protein, etc.), which results in mitochondrial damage and the opening of the mitochondrial permeability transition pore (mPTP), while at the same time the damaged mitochondria synthesize more radicals, which forms a cascade amplification. Exogenous supplement of NAC increases the ability of scavenging radicals, which decreases the level of radicals in mitochondria and restores the balance of radical synthesis and scavenging in mitochondrial damage (Thounaojam et al., 2011; Mantzarlis et al., 2017; Cadenas, 2018; Duan et al., 2020).

Our research found that NAC had a good therapeutic effect on hemorrhagic shock combined with seawater immersion in a rat model; nevertheless, some limitations need to be overcome in subsequent studies. For example, the present experiment was performed in rats, and relevant experiments need to be confirmed in large animals and human beings. Furthermore, the model of pressure-controlled hemorrhage is stable. Therefore, we used it to explore NAC in hemorrhagic shock combined with seawater immersion and found that NAC played a great role. However, regardless of location, most patients or the wounded are in a non-controlled hemorrhage condition. Hence, the model of pressure-controlled hemorrhage used in this experiment could not fully investigate the potential therapeutic effect of NAC on hemorrhagic shock combined with seawater immersion.

In addition, the anesthesia in this experiment lasted no less than 6 h. Whether prolonged anesthesia would influence the therapeutic effect of LR, which might cause a biased effect between the LR group and NAC group, was unclear. Similarly, heparin is not clinically used in patients with hemorrhagic shock, but in this experiment, heparin was added in rats to prevent blood clotting to keep the polyethylene catheter clear for drawing blood and measuring MAP.

Barbee et al. (2010) and de Vrij et al. (2014) pointed out that improper thawing without rectifying hypertonic dehydration and electrolyte disturbances may increase the metabolic rate, exacerbate organization hypertonic dehydration and metabolic acidosis, and could even lead to peripheral vascular expansion, further reduction of blood volume, lower blood pressure, and heart damage as a consequence. Therefore, we insisted firmly that stepwise rewarming should be taken into consideration in this study.

## Conclusion

Generally, our study discovered that hemorrhagic shock combined with seawater immersion could be deadly and led to a severe lethal triad and serious multi-organ dysfunction, which was associated with oxidative stress and impaired mitochondrial function. LR solution with the addition of NAC could improve animal survival, reverse acidosis and coagulopathy, and protect multi-organ function significantly via its antioxidant properties, and may have potential therapeutic applications as an adjunct in fluid resuscitation.

## Data Availability Statement

The original contributions presented in the study are included in the article/supplementary material, further inquiries can be directed to the corresponding authors.

## Ethics Statement

The animal study was reviewed and approved by the Laboratory Animal Welfare and Ethics Committee of Third Military Medical University.

## Author Contributions

TL and LL designed the study, obtained research funding, and took responsibility for the manuscript as a whole. YL, YZ, ZZ, HD, and DB performed the animal experiments. YL analyzed the data and drafted the manuscript. All authors read and approved the final manuscript.

## Conflict of Interest

The authors declare that the research was conducted in the absence of any commercial or financial relationships that could be construed as a potential conflict of interest.

## Publisher’s Note

All claims expressed in this article are solely those of the authors and do not necessarily represent those of their affiliated organizations, or those of the publisher, the editors and the reviewers. Any product that may be evaluated in this article, or claim that may be made by its manufacturer, is not guaranteed or endorsed by the publisher.
